# Supplementary material for: A Pilot Study on Oxidative Stress during the Recovery Phase in Critical COVID-19 Patients in a Rehabilitation Facility: Potential Utility of the PAOT® Technology for Assessing Total Anti-Oxidative Capacity
Source: Biomedicines. 2023 Apr 28;11(5):1308. doi: 10.3390/biomedicines11051308 (PMC10216138; doi:10.3390/biomedicines11051308)
Supplement: Supplementary file 1 [file biomedicines-11-01308-s001.zip › biomedicines-2124990-supplementary.pptx]

## Slide 1
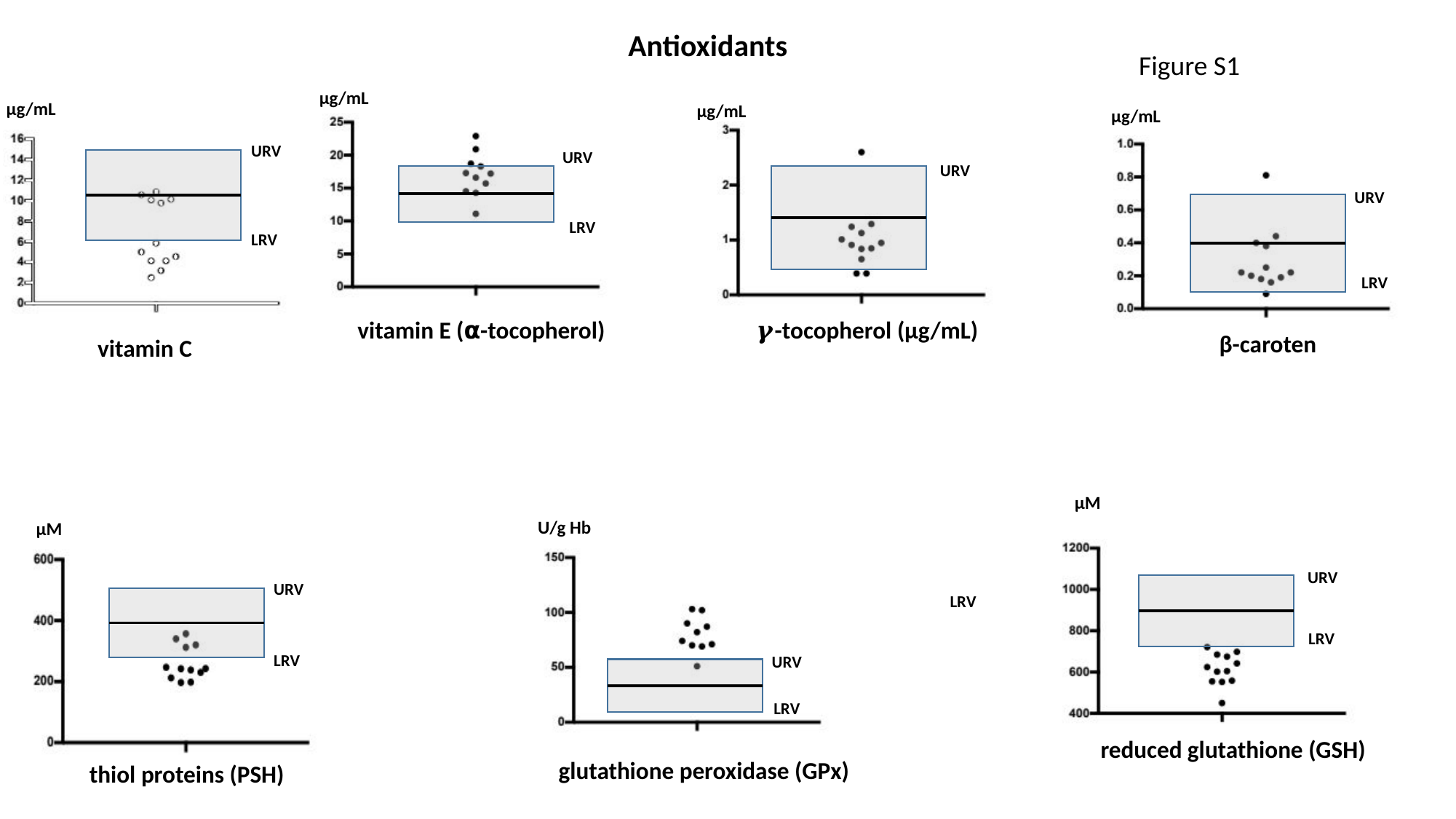

Antioxidants
Figure S1
µg/mL
µg/mL
URV
LRV
vitamin C
µg/mL
URV
𝜸-tocopherol (µg/mL)
LRV
µg/mL
URV
URV
LRV
LRV
vitamin E (⍺-tocopherol)
β-caroten
µM
reduced glutathione (GSH)
U/g Hb
glutathione peroxidase (GPx)
µM
thiol proteins (PSH)
URV
URV
LRV
LRV
URV
LRV

## Slide 2
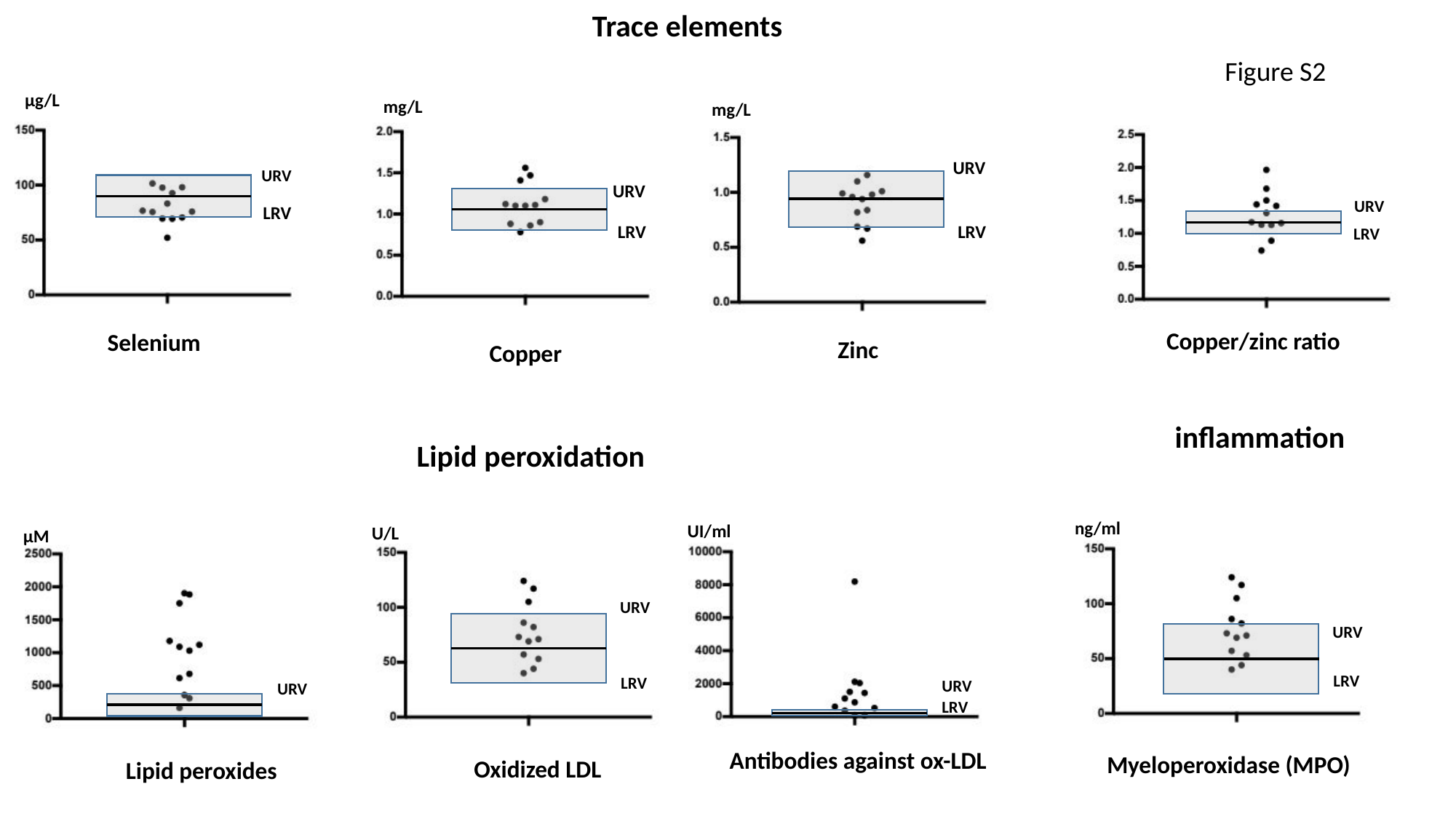

Trace elements
Figure S2
µg/L
mg/L
URV
LRV
Copper
mg/L
URV
URV
URV
LRV
LRV
LRV
Copper/zinc ratio
Selenium
Zinc
inflammation
Lipid peroxidation
ng/ml
UI/ml
U/L
µM
URV
URV
LRV
LRV
URV
URV
LRV
Antibodies against ox-LDL
Myeloperoxidase (MPO)
Oxidized LDL
Lipid peroxides

## Slide 3
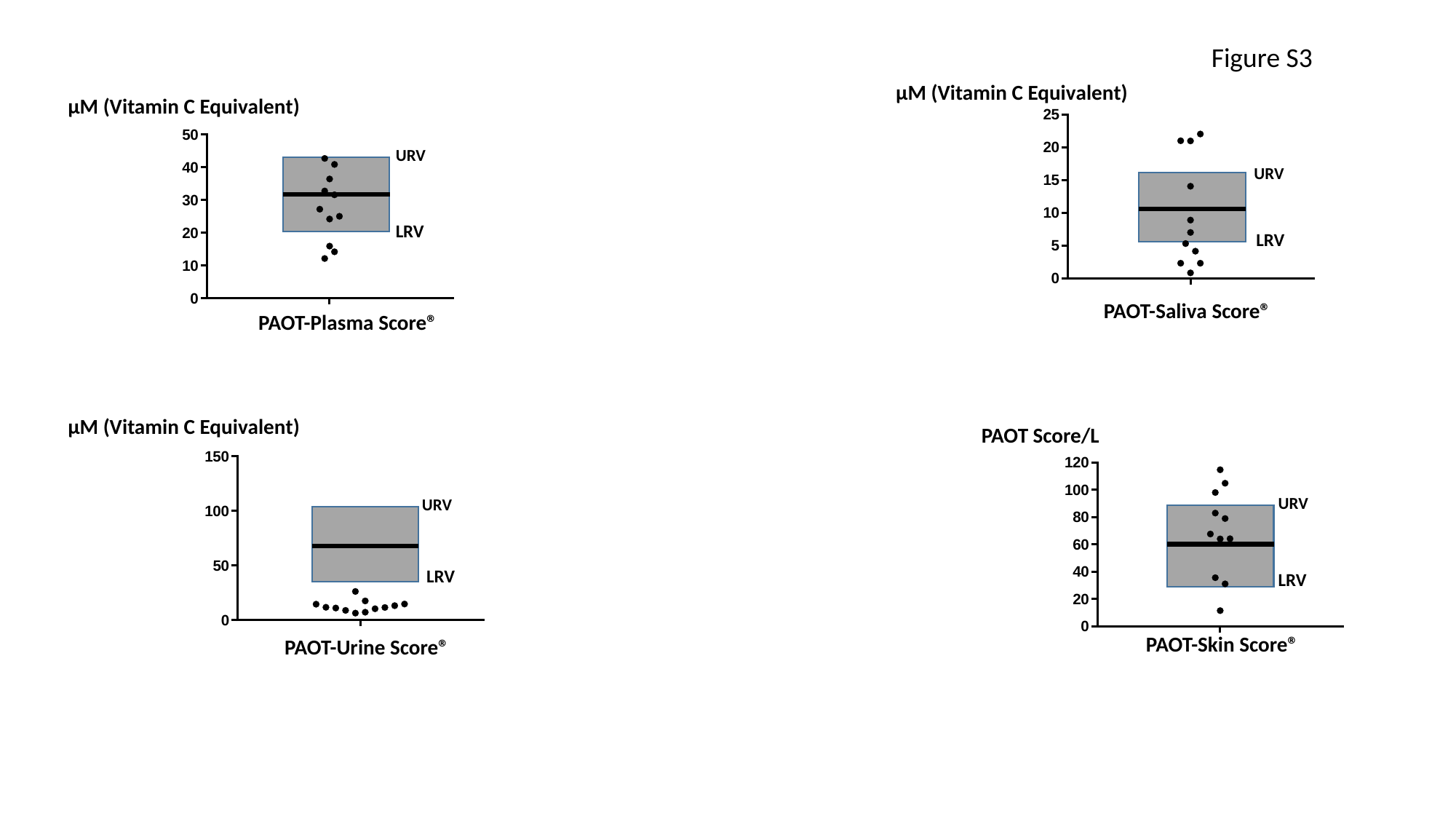

PAOT Score/L
Figure S3
µM (Vitamin C Equivalent)
µM (Vitamin C Equivalent)
URV
URV
LRV
LRV
PAOT-Saliva Score®
PAOT-Plasma Score®
µM (Vitamin C Equivalent)
PAOT Score/L
URV
URV
LRV
LRV
PAOT-Skin Score®
PAOT-Urine Score®
UNV
